# Buccal Silicone Granuloma Caused by the Dental Infection

**DOI:** 10.1155/2020/8834475

**Published:** 2020-11-03

**Authors:** Kazuhiro Murakami, Kazuhiko Yamamoto, Tsutomu Sugiura, Tadaaki Kirita

**Affiliations:** Department of Oral and Maxillofacial Surgery, Nara Medical, University, Kashihara City, Nara, Japan

## Abstract

The facial injection of liquid silicone is performed for cosmetic purposes. The use of injectable fillers in facial procedures has become extremely popular over the past decade. Most procedures are performed in the perioral, periocular, and cheek areas of middle-aged women. Even though silicone is biologically inert, its injection can result in the formation of granulomas. Silicone granulomas can result from an inflammatory or autoimmune tissue response. However, the development of silicone granulomas secondary to dental infection has not yet been reported. We report a case of a 73-year-old woman with a right buccal silicone granuloma that developed following a dental infection. Ultimately, this case healed completely after the surgical removal of all lesions. Silicone in the facial region may become infected by a dental infection, and infective silicone develops granulomas and cellulitis. In the context of cosmetic facial silicone injections, it is necessary to improve oral hygiene prior to dental treatment and to maintain a healthy oral environment after surgery. In some cases, surgical treatment using an intraoral approach is effective.

## 1. Introduction

The use of injectable fillers in facial procedures has become extremely popular over the past decade, with most procedures performed in the perioral, periocular, and cheek areas of middle-aged women [1]. It is aimed at smoothening out wrinkles or creases and at producing an artificial augmentation of lip or cheek volume for cosmetic and rejuvenation purposes [2].

The advantages of liquid silicone implantation include its low cost, stable chemical structure, and low degree of tissue reactivity [3]. However, even though it is biologically inert, silicone injection can result in the formation of granulomas [4]. The severity and frequency of granuloma formation, as well as other immunologic complications, will likely increase if injections are administrated by incautious or unqualified, perhaps unlicensed, practitioners using non-medical-grade silicone [1, 5]. Regardless of the origin and mechanism of silicone dissemination, silicone granulomas can result from an inflammatory or autoimmune tissue response [6]. However, the development of silicone granulomas due to dental infection has not yet been reported.

We report a case of a 73-year-old woman with a right buccal silicone granuloma developed by the dental infection.

## 2. Case Report

**Figure 1:**
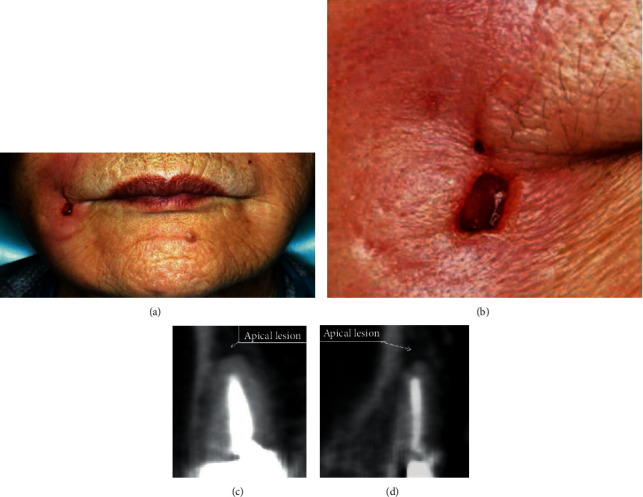
(a) Photograph of the front of the face taken at the first visit. Diffuse swelling of the right cheek was seen. A fistula of about 5 mm was present near the corner of the mouth. (b) Photograph around the fistula. Pus was discharging from the fistula. (c) Cone beamed computed tomography (CBCT) image at the causal tooth in coronal image. (d) CBCT image at the causal tooth in sagittal image. There was an apical lesion of about 3 mm in the root apex of the right maxillary second premolar.

**Figure 2:**
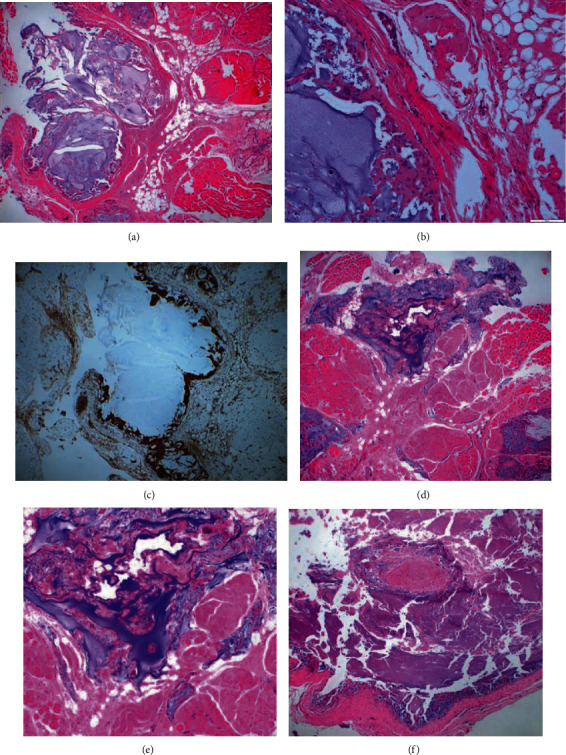
Histological findings from the first surgery: (a) histological examination of the resected fistula indicated the mucus-like deposition, the formation of mucous-like nodules, and the infiltration of foreign body giant cells in fat, striated muscle, and fibrous tissue, with mild calcification [hematoxylin-eosin (H-E) stain ×25]. (b) Calcification was present, many fiber cells were observed around it, and vacuolated cells and foreign body giant cells were seen [H-E stain ×100]. (c) Vimentin stain was positive for stromal cells and fibrocytes. Histopathological diagnosis was suspected of mucin-producing odontogenic tumor and mucinous adenocarcinoma, among other diagnoses. Histological findings from the second surgery: (d) histological examination of the removal tumor showed foreign body giant cell, vacuolated cells, fibrous tissue, and striated muscle around calcifications [H-E stain ×25]. (e) H-E stain ×100. (f) Silicone particles, foreign body giant cell, and fibrotic tissue of silicone granuloma [H-E stain ×25].

**Figure 3:**
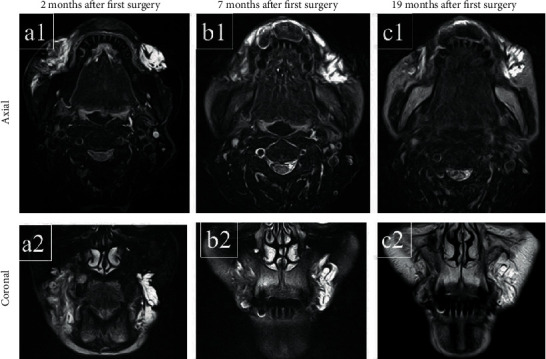
MRI findings. (a1, a2) Axial and coronal images of T2-weighted image of fat suppression two months after the first surgery. Irregular high signal intensity areas under the subcutaneous tissue on both cheeks and high signal intensity areas were widely distributed on the right side. (b1, b2) Axial and coronal images of T2-weighted image of fat suppression seven months after the first surgery. The images showed a reduction of the right nodule compared with those two months after the first surgery. (c1, c2) Axial and coronal images of T2-weighted image 19 months after the first surgery. Unclear boundary lesions showed the high signal area was recognized, and foreign body-like liquid silicone injection was suspected again. The above high signal area on the buccal region was shrinking over time. These findings should develop by loss of silicone from the fistula. The high signal area on the left buccal region had not changed.

A 73-year-old woman was referred from a private dentist to our department for swelling of the right maxillary premolar buccal gingiva and diffuse swelling of the right cheek. Excision and drainage of the cheek region had been performed with cosmetic surgery a month earlier. Her past medical history was unremarkable except for hypertension. There was a fistula of about 5 mm on the right cheek 10 mm above the oral angle, and pus was discharging from this fistula (Figures 1(a) and 1(b)). Cone beamed computed tomography (CBCT) imaging showed an apical lesion of the second premolar (Figures 1(c) and 1(d)). A bougie was inserted through the fistula and advanced, and it finally reached the apical region of the right upper second premolar. Based on these findings, the lesion was diagnosed as right buccal cellulitis and orofacial external fistula due to apical periodontitis of the right upper second premolar. According to this clinical diagnosis, antibiotics [cefcapene pivoxil (CFPN-PI), 300 mg/day] were administered for three days, and root canal treatment of the right upper first and second premolar was performed. Bacterial examination of pus from the fistula revealed gram-positive *α-streptococcus* (resident bacteria). The administration of antibiotics and root canal treatment improved the swelling of the right cheek. After the root canal treatment was completed, the external orofacial fistula was removed, and apicoectomy of the right upper first and second premolar was performed. Histological examination of the resected fistula indicated that there were mucus deposition, mucous nodule formation, and foreign body giant cell infiltration in the fat, striated muscle, and fibrous tissue, with mild calcification. AE1/AE3 staining was negative, and no epithelial components were observed. Vimentin staining revealed many positive stromal cells. Histopathological diagnosis suspected a mucin-producing odontogenic tumor or mucinous adenocarcinoma, among other diagnoses (Figures 2(a)–2(c)). Her symptoms had disappeared two months after the surgery. However, swelling of the right cheek and fistula formation developed again at the same site, and bacterial examination and magnetic resonance imaging (MRI) were performed. Bacteriological examination revealed the *Streptococcus anginosus* group. The MRI findings were irregular high signal intensity areas under the subcutaneous tissue on both cheeks on T2-weighted images of fat suppression, and high signal intensity areas were widely distributed on the right side (Figure 3 (a1) and (a2)).

**Figure 4:**
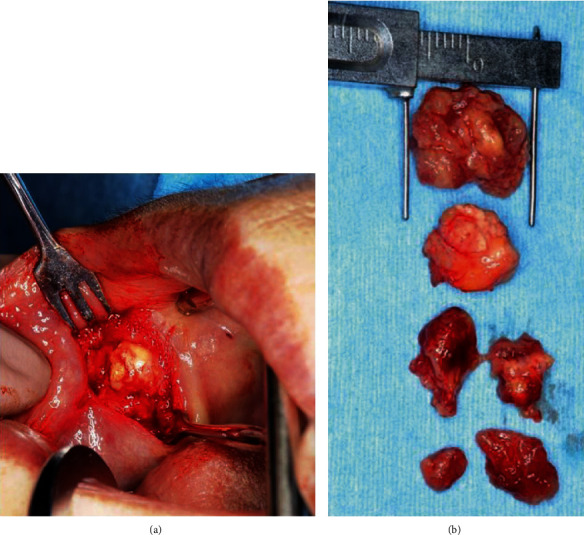
(a) Intraoperative photograph. (b) Photograph of the removed lesions. Most of the silicone granulomas containing the mass of silicone were removed.

**Figure 5:**
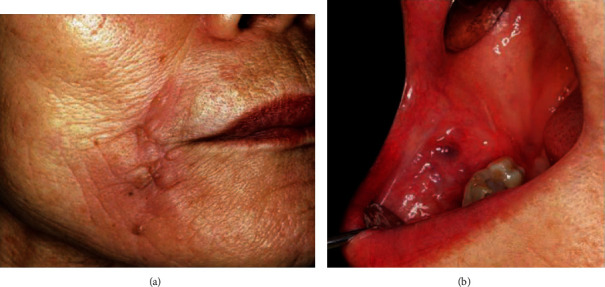
(a) Extraoral photograph three months after the second surgery. (b) Intraoral photograph three months after the second surgery. Although a slight scar formation was observed around the region where the fistula was present, the healing was completed.

We asked her if she had a past history of injection of any foreign body, but she denied having had liquid silicone injections. Since the *Streptococcus anginosus* group was sensitive for CFPN-PI, it was administered for 28 days. The swelling of the right buccal region disappeared, and there was a reduction of the fistula. However, since a small amount of pus-like effusion was observed from the fistula, minocycline ointment was injected subcutaneously into the fistula. The symptoms disappeared after treatment, but a single subcutaneous nodule, approximately 10 × 10 mm in size, remained in the cheek, and an extremely small quantity of clear effusion flowed from the fistula. Since the MRI showed a reduction of the nodule six months later, we continued follow-up (Figure 3 (b1) and (b2)). One year later, a swelling with fluctuation developed in the right cheek again, and puncture aspiration of pus was performed. The bacterial examination demonstrated methicillin-sensitive *Staphylococcus aureus* (MSSA), so CFPN-PI was administered. The MRI showed unclear boundary lesions in the high signal area in T2-weighted images and the low signal area in T1-weighted images, and a foreign body-like liquid silicone was suspected again (Figure 3 (c1) and (c2)). When we asked about her past medical history again, the patient revealed that liquid silicone had been injected into her cheeks eight years earlier to remove nasolabial folds. Removal of the foreign body was planned for radical treatment. Under local anesthesia, all connective tissue and granuloma, including the silicone from the buccal mucosa, were removed (Figures 4(a) and 4(b)). In the process, bleeding was observed from the upper lip branch of the facial artery, so hemostasis was performed by ablation, and the operation was completed. Histopathologically, the fibrotic hyperplasia was observed in the striated muscles and subcutaneous tissues, and a foreign body granuloma with liquid silicone and foreign body giant cells was identified. Histopathologic diagnoses were the silicone, silicone granuloma, and scar (Figures 2(d) and 2(e)). After the operation, she had no complications, such as facial paralysis or facial deformity (Figures 5(a) and 5(b)). The postoperative course was uneventful, and there were no symptoms for one year after surgery.

## 3. Discussion

Silicone (polysiloxane) is known widely by general, cosmetic, and surgical dermatologists and is available in gel-filled implants as well as in liquid and solid forms. Liquid and gel implants may be used for tissue augmentation (e.g., breast) and as esthetic fillers [3]. Mostly performed in the perioral, periocular, and cheek areas of middle-aged women, the aim is to smoothen out wrinkles or creases and to produce an artificial augmentation of the lip or cheek volume for cosmetic and rejuvenation purposes [2].

The injection of artificial substances began in the 1950s, and liquid silicone was used up until the early 1960s. However, the U.S. Food and Drug Administration (FDA) banned silicone injections in 1965 due to complications following injection. However, in 1994, purified and sterilized high-viscosity silicone oil made by Adatomed (Munich, Germany) was introduced for ophthalmologic use and was approved by the U.S. FDA for that use. The majority of complications include granulomas, nodularity, migration, and chronic cellulitis [7]. Even though silicone is biologically inert, its injection can result in the formation of granulomas. Silicone granulomas were first described in 1964 as a rare complication that was, and continues to be, a diagnostic challenge. The severity and frequency of granuloma formation, as well as other immunologic complications, will likely increase if injections are administered by incautious or unqualified, perhaps unlicensed, practitioners using non-medical-grade silicone [1]. Although there are several case reports of complications arising from silicone use, the amount of silicone was frequently related in the past with the appearance of granulomas. Impurity from injected liquid silicone can be seen microscopically as translucent, birefringent foreign bodies within the cytoplasm of giant cells [3].

The likelihood of the appearance of silicone granulomas is greater with large volumes of injection. There are reports that pure silicone, administered in small amounts, does not cause granulomas [5]. Liquid silicone is administered with the microdroplet technique, in which very small amounts are injected using a serial puncture technique [1]. Given that there are significant potential benefits to purified, high-viscosity, injectable silicone oil if used correctly, it is important to elucidate the factors that contribute to complications [1]. Although detailed information about the liquid silicone administration method used in this case was not available, it was supposed that the appropriate administration method had probably been performed based on the patient's interview. In this case, it was supposed the silicone granulomas were developed not by immune response reaction of the silicone but by the infection of it. As a cause of that, the cellulitis on the right cheek was initially occurring, a bougie inserted through the fistula reached the apical region of the right upper second premolar, and bacterial examination of pus with silicone particles from the fistula revealed oral resident bacteria. Furthermore, the liquid silicone was injected into the cheeks on both sides; however, cellulitis, fistula, and granuloma formation were observed only on the right side. Therefore, if the silicone injection was done properly, this case was considered to have been caused by a dental infection.

Travis et al. reported the classification into three types: (1) inflow into soft tissues (granulomatous reaction), (2) uptake into lymph nodes, and (3) inflow into blood vessels (human adjuvant disease) [8]. Regardless of the origin and mechanism of silicone dissemination, silicone granulomas can result from an inflammatory or autoimmune tissue response. A systemic manifestation of the inflammatory response to adjuvants, such as silicone, has been termed autoimmune/inflammatory syndrome induced by adjuvants [6]. In this case, no systemic symptoms, such as collagen disease and multiple organ lesions, were observed, and only local inflammation was observed. Silicone granulomas are as prevalent as they have always been, but there is no consensus regarding their treatment. Treatment can be difficult and unsuccessful in many cases. Clinicians should try to individualize treatment for each patient. If surgical excision is deemed necessary, significant scarring can be expected because the silicone itself and the granulomas can migrate throughout multiple layers of soft tissue, often necessitating the removal of thick sections of tissue. In some patients, granulomas will resolve spontaneously without treatment. The more common treatments include systemic and local steroids, minocycline, 5-fluorouracil, isotretinoin, and, for localized granuloma formation, surgical resection. While success has been achieved with steroids, relapse often occurs after a steroid taper. Tetracycline antibiotics, especially minocycline, have been used successfully for their anti-inflammatory and antigranulomatous properties, as well as their mycobacterial coverage. Surgical resection is a good option for localized granulomas, although the resected tissue must be replaced to fill the dead space and minimize postoperative esthetic deformity [1, 9]. The rationale for the administration of minocycline in granulomatous tissue reaction is its anti-inflammatory, immunomodulating, and antigranulomatous effects [9]. After removal of the apical lesion and the external fistula, the facial fistula remained from the infected silicone lesion, so minocycline ointment was administered through the fistula. In this case, the minocycline was locally administered through the fistula and the symptoms improved temporarily, but inflammation occurred again and MSSA was detected in the pus from the fistula. Although it was reported that oral administration of minocycline (100 mg once daily) was effective in a previous report [9], it was unclear whether local administration of minocycline was effective in this case.

The silicone scattered from this region was washed away from the fistula, and the lumped silicone changed the silicone granuloma. It was considered that most of the silicone in the region could be removed, and the healing was actually achieved by removing this granuloma. Facial deformity did not occur in the removal region because it was considered that only a small amount of silicone, which was used for the purpose of smoothing out wrinkles, had been injected. The surgical intervention proved to be effective in this case.

Although artificial materials are injected into many regions of the body in cosmetic surgery, the injection of nonabsorbable materials into the facial region has a greater tendency to become infected than other body areas because the facial region is adjacent to the oral tissue. In particular, since the thin facial muscles are present between the subcutaneous tissues of the cheeks and chin and the oral mucosa, an oral infection easily develops into a subcutaneous silicone infection. In esthetic treatments, it is important to have good oral hygiene, and the condition of the oral region, including the teeth and gingiva, needs to be hygienic.

The histologic diagnosis of silicone granuloma is easily interpreted in most cases. However, as in the present case, the patient may fail to report a history of cosmetic surgery. Without a complete clinical history, there is an increased risk of histopathologic misinterpretation [10]. The histology is characterized by multinucleated giant cells as a foreign body reaction. Vacuoles corresponding to the filler material are frequently found, particularly with silicone. With other products, the filler material may not be detected. The differential diagnosis should include erysipelas, allergic contact dermatitis, facial edema with eosinophilia, cheilitis glandularis apostematosa, Ascher's syndrome, orofacial granulomatosis, Crohn's disease, Melkersson-Rosenthal syndrome, and sarcoidosis. Likewise, cutaneous leishmaniasis, leprosy, and tuberculosis can also present as granulomatous inflammations of the skin.^4^ Gonçales et al. [10] reported that when the patient denies previous cosmetic procedures, these cases can be misdiagnosed as malignant tumors, such as low-grade liposarcoma. Since the facial silicone granulomas related to esthetics, the patient's pathological history is often concealed. Even in this case, the initial clinical diagnosis, which included mucin-producing odontogenic tumor and mucinous adenocarcinoma, was based on the first histopathologic examination because the patient did not reveal her history. It has been reported that it is necessary to listen closely to the interview [10]. Although the silicone injection was performed properly in this case, it can be considered that the formation of the silicone granuloma was associated with abscess and fistula formation due to an unexpected dental infection. For patients with suspected dental infections, it is desirable for the infection to be resolved and the dental treatment to be completed before silicone injections are performed. Also, it is necessary to maintain good intraoral hygiene after the injections are completed.

## 4. Conclusion

The facial injection of liquid silicone is performed for cosmetic purposes, but the silicone may become infected by a dental infection, and the infective silicone may develop granulomas and cellulitis. It is necessary to complete preoperative dental treatment to improve oral hygiene and to maintain a clean oral environment after surgery. In some cases, surgical treatment using an intraoral approach is effective.
